# Reduced Mitochondrial DNA Copy Number and Telomere Length in Essential Tremor Patients: Evidence from an Age- and Sex-Adjusted Cross-Sectional Case–Control Study

**DOI:** 10.3390/ijms27125275

**Published:** 2026-06-10

**Authors:** Monica Gagliardi, Alessia Felicetti, Radha Procopio, Antonio Augimeri, Costanza Maria Cristiani, Maurizio Morelli, Giuseppe Pedullà, Andrea Quattrone, Grazia Annesi, Aldo Quattrone

**Affiliations:** 1 Neuroscience Research Center, Magna Graecia University, 88100 Catanzaro, Italy; radha.procopio@unicz.it (R.P.); costanza.cristiani@unicz.it (C.M.C.); an.quattrone@unicz.it (A.Q.); quattrone@unicz.it (A.Q.); 2 Department of Medical and Surgical Sciences, Institute of Neurology, Magna Graecia University, 88100 Catanzaro, Italy; alessia.felicetti@studenti.unicz.it (A.F.); m.morelli@unicz.it (M.M.); giuseppe.pedulla001@studenti.unicz.it (G.P.); 3 Biotecnomed S.C.aR.L., 88100 Catanzaro, Italy; a.augimeri@unicz.it; 4 Institute for Biomedical Research and Innovation, National Research Council, 87050 Cosenza, Italy; grazia.annesi@cnr.it

**Keywords:** essential tremor, mitochondria, telomere

## Abstract

Essential tremor (ET) is a common movement disorder increasingly recognized as a complex syndrome with neurodegenerative features. While mitochondrial dysfunction and cellular aging are implicated in several neurodegenerative diseases, their role in ET remains unexplored. To investigate mitochondrial DNA copy number (mtDNA-CN) and telomere length (TL) in patients with ET and evaluate their potential as biomarkers of mitochondrial dysfunction and biological aging. In this cross-sectional case–control study, 68 ET patients (median age 66 years; 64.7% male) and 62 healthy controls (median age 70 years; 54.8% male) were enrolled. Relative mtDNA-CN and TL were quantified by quantitative PCR, measuring mitochondrial *ND1* gene levels and telomere-to-single-copy gene (T/S) ratio, respectively, both normalized to β-actin. Associations with disease status were assessed using age- and sex-adjusted multivariable linear regression on log_2_-transformed data, with statistical significance defined as *p* < 0.05 after false discovery rate (FDR)-corrected Wald tests. Receiver operating characteristic (ROC) and effect size (Cohen’s d) analyses were performed. ET patients showed significantly reduced mtDNA-CN (β = −2.785, 95% CI −3.700 to −1.869; *p_FDR_* = 2.53 × 10^−9^) and TL (β = −2.073, 95% CI −2.758 to −1.388; *p_FDR_* = 3.00 × 10^−9^), corresponding to ~6.9-fold and ~4.2-fold reductions, respectively. Age- and sex-stratified analyses confirmed consistent reductions, more pronounced in older individuals. Both biomarkers showed good discriminatory performance (mtDNA-CN: AUC = 0.83, 95% CI: 0.75–0.90; TL: AUC = 0.76, 95% CI: 0.68–0.85) and large effect sizes (Cohen’s d = |1.192| and |1.058|), respectively. Reduced mtDNA-CN and TL support the involvement of mitochondrial impairment and accelerated cellular aging in ET and may represent accessible peripheral biomarkers and provide a basis for future longitudinal and mechanistic investigations.

## 1. Introduction

Essential tremor (ET) is one of the most common neurological and movement disorders worldwide. Affecting approximately 0.9% of the general population and more than 4–5% of individuals older than 65 years [1]. Epidemiological data indicate that prevalence increases markedly with age, rising by nearly 74% with each advancing decade after 40 years [1]. The highest prevalence rates have been reported in elderly populations from Spain, Turkey, China, and the United States, whereas lower crude estimates have been observed in younger populations from countries such as Nigeria, Bangladesh, and Papua New Guinea [1]. These geographic differences are likely influenced by demographic structure, age distribution, methodological variability across studies, and underdiagnosis, rather than by a single country-specific risk factor. Importantly, population-based investigations indicate that 60% to more than 90% of affected individuals may remain undiagnosed, highlighting the underrecognized nature of ET and the limitations of current screening strategies [2,3].

Although traditionally considered a benign and monosymptomatic condition, ET is now increasingly recognized as a complex syndrome frequently associated with non-motor features, including cognitive impairment, neuropsychiatric symptoms, and gait disturbances [3,4]. Indeed, a wide spectrum of non-motor manifestations, such as anxiety, depression, and sensory alterations, has been consistently reported, supporting a broader conceptualization of essential tremor as a multisystem disorder rather than a purely motor condition [5]. Longitudinal clinical and neurophysiological studies further demonstrate that ET progression is characterized by the spread of tremor to multiple body segments and the emergence of additional “soft” neurological signs, reinforcing the dynamic and evolving nature of the disease phenotype [6]. Moreover, clinical and pathological overlaps with other neurodegenerative disorders, such as Parkinson’s disease, suggest that ET may share common underlying biological mechanisms [7]. Epidemiological and genetic studies further support this overlap, demonstrating an increased risk of Parkinson’s disease in ET patients and shared susceptibility pathways between the two conditions [8,9]. This overlap has contributed to the hypothesis that essential tremor may represent a syndrome encompassing multiple underlying disorders rather than a single nosological entity [10].

The etiology of ET remains incompletely understood and is likely multifactorial. A strong genetic contribution is supported by familial aggregation and high heritability; however, its genetic architecture is highly heterogeneous, involving both rare variants with potentially large effects and common variants with low penetrance [11]. Large genome-wide association studies have identified susceptibility loci explaining part of ET heritability and highlighted enrichment in cerebellar and axonogenesis-related pathways [12]. More recent large-scale genetic studies have identified novel disease-associated genes, such as *SLC38A6*, whose dysfunction affects amino acid transport, Purkinje cell excitability, and cerebellar circuitry, further supporting a central role of cerebellar pathology in ET [13]. Recent sequencing studies have identified candidate genes involved in diverse biological pathways, including γ-aminobutyric acid (GABA)-ergic neurotransmission, calcium signaling, protein homeostasis, and mitochondrial function, supporting the notion that ET represents a spectrum of disorders rather than a single disease entity [14]. Additional genetic mechanisms have been proposed, including repeat expansion disorders such as GGC expansions in the *NOTCH2NLC* gene, which segregate with disease in familial cases and are associated with more severe phenotypes, further highlighting the marked genetic heterogeneity of ET [15]. However, most identified variants remain rare or population-specific, and no single causative gene has been consistently replicated across different cohorts [16].

In parallel, converging evidence implicates cerebellar dysfunction and alterations in cerebello-thalamo-cortical circuits, with abnormalities in Purkinje cells and inhibitory neurotransmission contributing to tremor generation [7]. Neurophysiological and pathological studies indicate that ET may arise from abnormal oscillatory activity within central motor networks, particularly involving cerebellar circuits, rather than from a single structural lesion [17]. Postmortem and neurochemical investigations have further demonstrated impaired GABAergic neurotransmission, including reduced GABA receptor expression in the dentate nucleus, supporting a key role for inhibitory dysfunction in tremor pathogenesis [18].

Within this framework, increasing attention has been directed toward molecular mechanisms related to mitochondrial homeostasis and cellular aging. Mitochondria play a central role in neuronal function, and mitochondrial DNA copy number (mtDNA-CN) has emerged as a proxy of mitochondrial activity and bioenergetic capacity [19]. Alterations in mtDNA-CN have been described in several neurodegenerative disorders, reflecting either compensatory responses or underlying mitochondrial dysfunction [20,21,22]. However, evidence directly linking mitochondrial alterations to ET remains limited but biologically relevant. Yoo et al. reported multiple mtDNA deletions and abnormalities in mitochondrial oxidative phosphorylation complexes in ET patients, suggesting impaired mitochondrial integrity and bioenergetic dysfunction as possible contributors to disease pathophysiology [23]. In addition, the mitochondrial serine protease *HTRA2* p.G399S mutation was identified in a family with ET and Parkinson disease overlap, further supporting the involvement of mitochondrial quality-control pathways in tremor-related neurodegeneration [24].

However, no disease-specific studies have systematically evaluated peripheral mtDNA copy number in ET, and telomere length (TL) remains largely unexplored in clinically characterized ET cohorts. Telomere length, a marker of biological aging and cellular senescence, has been investigated in other neurodegenerative disorders, particularly Parkinson’s and Alzheimer’s disease, but its relevance to ET is still unclear [25,26,27,28,29]. Notably, mitochondrial dysfunction and telomere attrition are biologically interconnected processes, both influenced by oxidative stress and capable of contributing to neuronal vulnerability [30,31]. Given the evidence supporting neurodegenerative and aging-related mechanisms in ET, investigating mtDNA-CN and TL may help clarify the contribution of mitochondrial dysfunction and biological aging to disease pathophysiology. By jointly assessing these biomarkers in ET patients and healthy controls, the present study addresses an important gap in the literature and explores their potential as accessible peripheral biomarkers of systemic bioenergetic impairment. In this context, the present work aimed to evaluate mitochondrial DNA copy number and telomere length in a cohort of 68 patients with essential tremor relative to 62 healthy controls in order to explore their potential involvement in disease-related biological aging and mitochondrial dysfunction. We hypothesized that ET patients would show reduced mtDNA-CN and shorter telomere length compared with healthy controls, reflecting systemic mitochondrial dysfunction and accelerated biological aging. We further hypothesized that these alterations would discriminate ET patients from controls and may therefore represent accessible peripheral biomarkers of disease-related biological changes.

## 2. Results

### 2.1. Subjects

A total of 130 subjects were recruited at the Neuroscience Research Center, University Magna Graecia of Catanzaro, Italy, for the assessment of mtDNA-CN and telomere length. In this cross-sectional case–control study, we enrolled 68 patients diagnosed with essential tremor (ET) and 62 healthy controls (HC). Age significantly differed between ET and HC groups (Mann–Whitney *U* test, *p* = 0.0086), whereas sex distribution did not (χ^2^ test, *p* = 0.3332). The groups were matched by sex, with ET patients having a median age of 66 years (64.7% male) and healthy controls of 70 years (54.8% male). Demographic characteristics of both groups are summarized in Table 1. The study was authorized by the Calabria Region Ethics Committee under protocol code 143 on 13 May 2024. All participants provided informed consent in accordance with the institutional review board requirements.

### 2.2. Assay Reproducibility and Statistical Validation

Mean intra-assay coefficients of variation (CVs) were 0.97% for ND1, 0.75% for β-actin, and 1.53% for telomere, indicating high analytical precision and reproducibility. Residual diagnostics indicated deviations from normality for both mtDNA-CN and telomere length (Shapiro–Wilk *p* < 0.001; Figures S1 and S2). Breusch–Pagan tests showed evidence of heteroskedasticity for both mtDNA-CN (*p* = 0.03924) and telomere length (*p* = 0.00727). Accordingly, all inferences were based on HC3 robust standard errors. Power analysis indicated that the ET versus HC comparison was adequately powered to detect moderate effect sizes, with a minimum detectable effect size of |d| = 0.496 at 80% power and |d| = 0.574 at 90% power.

### 2.3. Reduced mtDNA-CN and TL in Essential Tremor Patients

**Figure 1:**
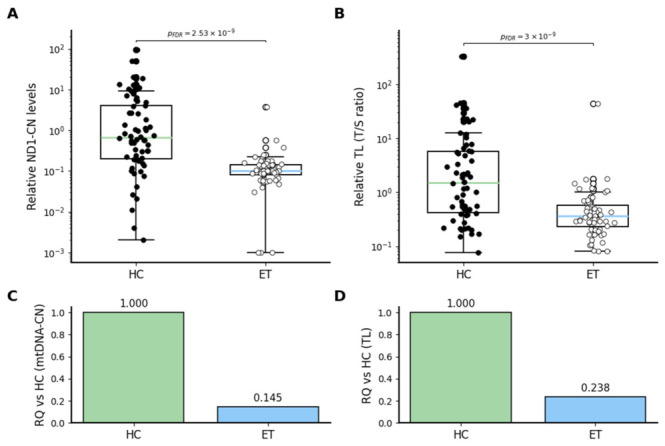
Comparison of mitochondrial DNA copy number (mtDNA-CN) and telomere length (TL) between HC and ET patients: (**A**) Relative *ND1*-CN levels (mtDNA-CN) and (**B**) relative TL (T/S ratio) are shown on a log_10_ scale. Boxplots display median and interquartile range with individual data points. Both mtDNA-CN and TL are significantly reduced in ET compared with HC (FDR-adjusted *p*-values reported). (**C**,**D**) RQ normalized to HC (set to 1.0) illustrates the magnitude of reduction in ET for mtDNA-CN and TL.

Relative *ND1*-CN levels were significantly reduced in ET compared to controls (Figure 1A; FDR-adjusted *p* = $2.53\;\times \;10^{-9}$). In the age- and sex-adjusted linear regression model, ET status was independently associated with lower log_2_-transformed *ND1*-CN (β = −2.785, 95% CI −3.700 to −1.869, *p* < 0.001), explaining 28.3% of the variance (R^2^ = 0.283). The standardized effect size was large (Cohen’s d = |1.192|), and only 8.8% of ET observations fell within the interquartile range of the HC distribution. Age- and sex-adjusted marginal means derived from the regression model corresponded to a relative quantification (RQ) value of 0.145 in ET relative to HC (Figure 1C), indicating an approximately 6.9-fold lower *ND1*-CN level in ET compared to the control group.

Relative telomere length (T/S ratio) was likewise significantly lower in ET compared with HC (Figure 1B; FDR-adjusted *p* = $3.00\times 10^{-9}$). After adjustment for age and sex, ET remained independently associated with reduced log_2_-transformed TL (β = −2.073, 95% CI −2.758 to −1.388, *p* < 0.001), with 22.6% of variance explained (R^2^ = 0.226). The effect size was large (d = |1.058|), and distributional overlap was greater than for mtDNA-CN, with 39.7% of ET values within the HC interquartile range. Model-derived adjusted RQ values indicated a relative 4.2-fold TL reduction in ET relative to HC (ET/HC RQ = 0.238; Figure 1D).

### 2.4. Association Analyses and Diagnostic Accuracy

In multivariable models, neither age nor sex was independently associated with mtDNA-CN (age: β = 0.0435, 95% CI −0.007 to 0.093, *p* = 0.088; male sex: β = 0.0890, 95% CI −0.838 to 1.016, *p* = 0.851) or telomere length (age: β = 0.0148, 95% CI −0.020 to 0.050, *p* = 0.407; male sex: β = 0.1831, 95% CI −0.575 to 0.941, *p* = 0.636).

**Figure 2:**
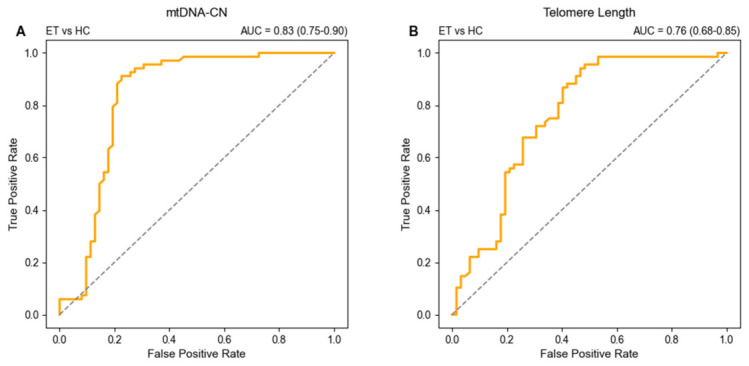
ROC analyses of mtDNA-CN and TL: (**A**) ROC curve evaluating the ability of whole blood mtDNA-CN to distinguish ET patients from HC, showing an AUC of 0.83 (95% CI: 0.75–0.90). (**B**) ROC curve for TL, measured as the T/S ratio, with an AUC of 0.76 (95% CI: 0.68–0.85). The dashed diagonal line represents the reference line for random classification (AUC = 0.5).

ROC analyses demonstrated robust discrimination of patients from healthy controls based on mtDNA-CN (AUC = 0.83, 95% CI 0.75–0.90; Figure 2A) and TL (AUC = 0.76, 95% CI 0.68–0.85; Figure 2B). Both biomarkers performed above chance level, with mtDNA-CN showing superior diagnostic performance relative to telomere length; however, this difference did not reach statistical significance (DeLong test, *p* = 0.153).

### 2.5. Age- and Sex-Specific Differences in mtDNA-CN and Telomere Length

**Figure 3:**
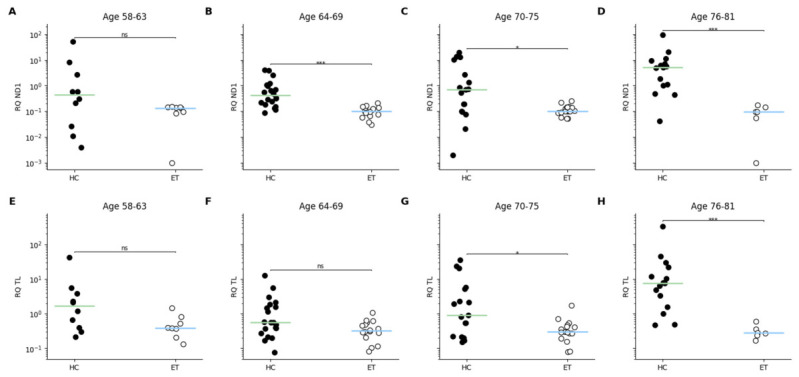
Age-stratified comparison of mtDNA-CN and TL between HC and ET patients. Relative mtDNA-CN (**A**–**D**) and relative TL (**E**–**H**) were compared between HC and ET patients across four age groups: 58–63 years (**A**,**E**), 64–69 years (**B**,**F**), 70–75 years (**C**,**G**), and 76–81 years (**D**,**H**). Data are shown on a log_10_ scale, with individual data points and group means indicated. Statistical significance is indicated as ns (not significant), * *p* < 0.05, and *** *p* < 0.001.

Age-stratified analyses demonstrated that the magnitude of group differences varied across age categories (Figure 3). For *ND1*-CN, no significant difference between ET and HC was observed in the 58–63 subgroup, whereas ET showed significantly lower levels in the 64–69, 70–75, and 76–81 strata. For TL, group differences were not significant in the 58–63 and 64–69 groups, but ET exhibited significantly shorter telomeres than controls in the 70–75 and 76–81 strata. Statistical power varied across age strata: in the 58–63 and 64–69 groups, observed effect sizes were moderate (Cohen’s d = −0.57 and −0.59) with corresponding power values of 0.31–0.34, whereas in the 70–75 and 76–81 strata, larger effect sizes were observed (Cohen’s d = −1.02 and −2.23) with power values of 0.89–1.00 for mtDNA and TL, respectively.

**Figure 4:**
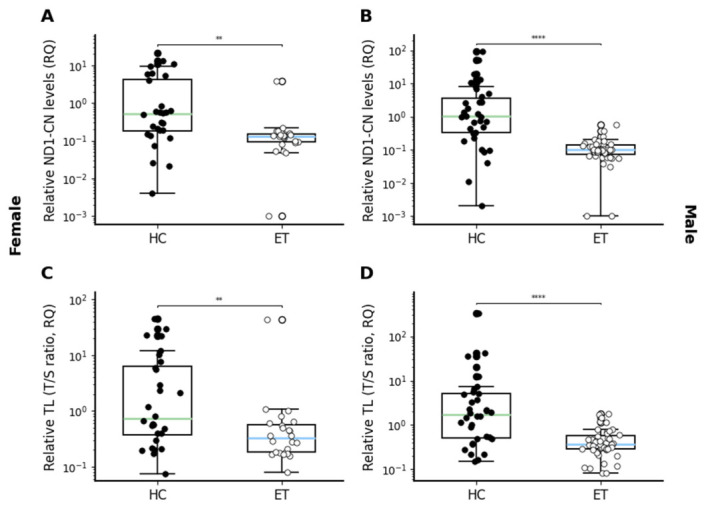
Sex-stratified analyses of mtDNA-CN and TL between healthy controls and ET patients. Relative *ND1*-CN levels (**A**,**B**) and relative TL (**C**,**D**) were analyzed between controls and ET patients after stratification by sex. Panels A and C show female participants, whereas panels B and D show male participants. Data are presented on a log_10_ scale, with individual values and boxplots indicating median and interquartile range. Statistical significance is indicated as ** *p* < 0.01 and **** *p* < 0.0001.

Sex-stratified analyses demonstrated significant reductions in both mtDNA-CN and telomere length in ET compared with HC in females and males (Figure 4). In females, ET exhibited significantly lower mtDNA-CN (FDR-adjusted *p* < 0.01) and TL (FDR-adjusted *p* < 0.01) relative to HC (Figure 4A,C), with effect sizes of −0.89 and −0.82 and corresponding power values of 0.88 and 0.82, respectively. In males, the magnitude of group differences was more pronounced, with significantly reduced mtDNA-CN and TL in ET compared with HC (FDR-adjusted *p* < 0.001; Figure 4B,D), corresponding to larger effect sizes (Cohen’s d = −1.36 and −1.27) and power values approaching 1.00.

### 2.6. Correlation Between mtDNA-CN and TL

**Figure 5:**
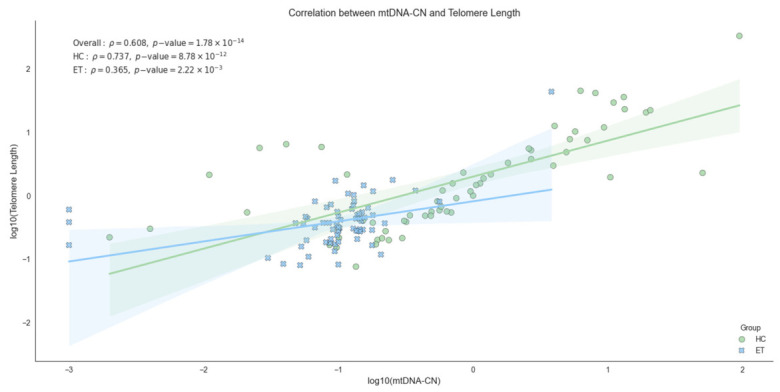
Correlation between mtDNA-CN and TL in the overall cohort, healthy controls (HC), and essential tremor (ET) patients. Scatter plots show log_10_-transformed mtDNA-CN and TL values. Colored lines represent linear regression models with 95% confidence intervals. Correlations were assessed using Spearman’s rank correlation coefficient. Abbreviation: *ρ*, rho.

Spearman correlation analysis revealed a significant positive correlation between mtDNA-CN and telomere length (TL) in the overall cohort (rho = 0.608, *p* = 1.78 × 10^−14^) (Figure 5). Group-stratified analyses showed significant correlations in both controls and ET patients, with a stronger correlation observed in HC subjects (HC: rho = 0.737, *p* = 8.78 × 10^−12^; ET: rho = 0.365, *p* = 2.22 × 10^−3^).

Association analyses confirmed these findings (Table 2). In the crude model, log_10_(mtDNA-CN) was significantly associated with log_10_(TL) (β = 0.562, 95% CI [0.410–0.714]; *p* = 4.31 × 10^−13^). After adjustment for age, sex, and disease group, the positive association persisted in the overall cohort (β = 0.510, 95% CI [0.313–0.708]; *p* = 3.93 × 10^−7^) and was also evident in the control group (β = 0.529, 95% CI [0.310–0.748]; *p* = 2.21 × 10^−6^). In ET, the regression coefficient was also positive, but the association was attenuated and did not reach statistical significance after adjustment (β = 0.319, 95% CI [−0.141–0.780]; *p* = 0.174). A formal interaction model showed no significant mtDNA-CN-by-group interaction (βinteraction = −0.257; *p* = 0.310), indicating no statistical evidence that the association differed between HC and ET.

## 3. Discussion

### 3.1. Mitochondrial and Telomeric Alterations in Essential Tremor

Our findings of significantly reduced mtDNA-CN in patients with ET support the presence of systemic mitochondrial alterations, potentially linked to impaired mitochondrial biogenesis and/or increased oxidative stress. Such mechanisms could plausibly contribute to Purkinje cell vulnerability and cerebellar dysfunction described in neuropathological studies of ET [32,33,34]. Similarly, telomere shortening is widely considered a marker of cellular aging and cumulative oxidative stress [35]. Given the close interplay between mitochondrial dysfunction and oxidative damage, the concurrent reduction in mtDNA-CN and TL may reflect converging biological pathways. Oxidative stress accelerates telomere attrition, while telomere dysfunction can, in turn, impair mitochondrial biogenesis through p53-mediated pathways, creating a bidirectional feedback loop between mitochondrial and telomeric integrity [30,31]. The parallel alterations observed in our cohort are therefore biologically coherent and suggest that essential tremor may involve systemic processes related to cellular aging and bioenergetic decline.

Consistent with this hypothesis, correlation and association analyses supported a significant positive relationship between mtDNA-CN and TL, suggesting that mitochondrial alterations and telomere shortening may represent interconnected biological processes related to cellular aging, replicative history, oxidative stress, or mitochondrial–nuclear homeostatic mechanisms. In group-specific adjusted models, this relationship was statistically robust in HC, whereas in ET it was positive but not significant after adjustment, suggesting that the mtDNA-CN–TL coupling may be less stable in the disease group. However, as formal evidence for a between-group difference was not observed, this subgroup pattern should be interpreted cautiously.

### 3.2. Age- and Sex-Related Biomarker Differences

Age-stratified analyses revealed more pronounced biomarker differences in older individuals, particularly for telomere length, which is consistent with the well-established progressive shortening of leukocyte telomeres with advancing age [36]. For mtDNA-CN, however, age-related patterns in whole blood must be interpreted cautiously due to platelet confounding. Because platelet counts decrease with age, particularly after ~50 years [37,38], older individuals contribute less platelet-derived mtDNA, producing an apparent decline in mtDNA-CN even if leukocyte mtDNA-CN remains unchanged [39]. However, age-dependent changes in blood cell composition must be considered alongside potential disease-related mechanisms. In this context, the greater reductions observed in older patients may not only reflect hematological shifts but also the cumulative impact of longer disease duration, which could contribute to progressive systemic mitochondrial alterations. Thus, although confounding by hematological composition must be considered, the age-dependent reduction in mtDNA-CN among ET patients is compatible with a disease-related mitochondrial component that becomes more evident with advancing age. Sex-stratified analyses demonstrated consistent reductions in both biomarkers across males and females, with somewhat stronger effects in males. While sex was not an independent predictor in regression models, this observation may warrant further investigation in larger cohorts to clarify potential sex-specific biological modulation.

### 3.3. Methodological Considerations and Study Limitations

Several limitations should be acknowledged when interpreting these findings. Different cell types exhibit substantial variability in mtDNA-CN, and differences in cellular composition can therefore introduce significant variability into tissue-level mtDNA-CN measurements [40]. Although whole blood provides a practical and minimally invasive source for biomarker assessment, circulating mtDNA-CN and telomere length may not accurately mirror mitochondrial and telomeric dynamics within the central nervous system. Consequently, the extent to which these peripheral alterations reflect cerebellar or broader neuronal changes remains uncertain. Moreover, potential confounding factors such as treatment exposure, metabolic status, lifestyle factors, and systemic inflammatory conditions were not systematically controlled and may have influenced the observed associations. Finally, the group differences are expressed as relative quantification measures, reflecting changes in the mtDNA:nDNA ratio relative to the HC calibrator rather than absolute mtDNA copies per cell. Given the ratiometric nature of blood mtDNA-CN and its sensitivity to leukocyte composition and platelet abundance, large fold changes should be interpreted cautiously, as they may partly reflect hematologic or cellular-mixture effects rather than proportional changes in mitochondrial respiratory capacity [40]. Accordingly, given the single-center Italian cohort and the relatively limited sample size, replication in independent, larger, and more diverse cohorts and validation using complementary approaches, such as flow cytometry-based cell sorting or detailed leukocyte phenotyping, are warranted to strengthen biological interpretation.

### 3.4. Conclusions

In conclusion, this study demonstrates that patients with essential tremor exhibit significantly reduced whole-blood mtDNA copy number and telomere length compared with healthy controls. The consistent alterations observed across analyses, together with their discriminatory performance, suggest that mtDNA-CN and TL may represent accessible peripheral biomarkers associated with disease-related biological changes in ET.

### 3.5. Future Perspectives

Future longitudinal and mechanistic studies are warranted to determine whether these biomarkers contribute to disease pathogenesis, progression, or therapeutic response.

## 4. Materials and Methods

### 4.1. Case Selection

This cross-sectional case–control study was carried out in accordance with the STROBE guidelines for cross-sectional studies. The completed checklist is provided as Table S2. Healthy controls were selected among older individuals and were excluded if they met any of the following criteria: a family history of essential tremor; neurological symptoms; current use of anticonvulsant medications; history of stroke or facial paralysis; or a diagnosis of major neurological disorders, including essential tremor, dementia, cognitive impairment, epilepsy, or sleep apnea. Patients were included if they fulfilled the International Parkinson and Movement Disorder Society (IPMDS) consensus criteria for essential tremor: an isolated bilateral upper-limb action tremor of at least 3 years’ duration, with or without tremor in other locations. Cases with dystonia, ataxia, Parkinsonism, isolated focal tremor, task- or position-specific tremor, orthostatic tremor, sudden onset, or stepwise deterioration were excluded.

### 4.2. Genetic Analysis

Genomic DNA from the whole blood of ET patients and HC was isolated using Maxwell^®^ RSC Blood DNA kit AS1880 (Promega, Madison, WI, USA), followed by the assessment of mtDNA-CN and TL via real-time quantitative PCR (qPCR) with SYBR Green chemistry. Each reaction had a total volume of 10 μL, containing 2 μL genomic DNA and 5 μL SYBR™ Green Universal Master Mix (Thermo Fisher Scientific, Waltham, MA, USA, catalog number: 4309155). β-actin (*ACTB*) served as the reference single-copy gene for ΔΔCt normalization, enabling the calculation of both the telomere-to-single-copy gene (T/S) ratio and relative *ND1* gene levels. All PCRs were conducted in duplicate on a QuantStudio™ 5 Real-Time PCR System (Applied Biosystems, Waltham, MA, USA) according to the MIQE guidelines [41]. *ACTB* and *ND1* were analyzed on the same plate per sample, while telomere measurements were carried out by maintaining the same well positions across runs. Thermal cycling conditions for *ND1* and *ACTB* PCRs were as follows: 1 cycle at 95 °C for 10 min, followed by 40 cycles of 95 °C for 10 s and 60 °C for 30 s. For telomere length assessment, PCRs were conducted at 95 °C for 10 min, then 40 cycles of 95 °C for 15 s and 56 °C for 60 s. Primer sequences are listed in Table S1. Relative quantification was calculated using the $2^{-∆∆Ct}$ method, under the assumption of comparable and high amplification efficiencies. Validation experiments demonstrated optimal and closely matched efficiencies for ND1 (109.08%), telomere (99.37%), and β-actin (104.52%) (Figure S3). Coefficients of variation (CVs) were calculated for ND1, β-actin, and telomere assays.

### 4.3. Statistical Analysis

All statistical computations were performed in Python (version 3.13.9, Python Software Foundation, Wilmington, DE, USA). Data distribution was assessed using the Shapiro–Wilk test, and homogeneity of variance was evaluated using Levene’s test. Continuous variables are reported as median and interquartile range (Q1–Q3), whereas categorical variables are expressed as counts and percentages. Continuous variables were compared using Student’s *t*-test or the Mann–Whitney U test and categorical variables using χ^2^ or Fisher’s exact test, as appropriate. A *p*-value < 0.05 was considered statistically significant. Relative mtDNA-CN (*ND1*) and telomere length (T/S ratio) were measured by qPCR and expressed as relative quantification (RQ) values using the $2^{-∆∆Ct}$ method. For regression-based inference, RQ values were log_2_-transformed to stabilize variance and approximate normality before modeling. Associations with disease status (HC, ET) were examined using multivariable linear regression models, including age and sex as covariates, implemented with the statsmodels package v.0.14.4. Model assumptions were assessed by visual inspection of Q-Q plots and residuals versus fitted values. Residual normality was formally evaluated using the Shapiro–Wilk test, and heteroskedasticity was assessed using the Breusch–Pagan test. Statistical inference for the ET versus HC effect was based on two-sided Wald tests derived from linear regression models fitted with HC3 heteroskedasticity-robust standard errors. To control for multiple testing, *p*-values were adjusted using the Benjamini–Hochberg false discovery rate (FDR) procedure, with adjusted *p*-values < 0.05 considered statistically significant. Minimum detectable effect sizes (Cohen’s d) were estimated for the ET versus HC comparison based on sample size, α = 0.05, and 80–90% power. Distributional overlap was defined as the proportion of disease-group observations within the healthy control interquartile range. Diagnostic performance was assessed by receiver operating characteristic (ROC) analysis. Area under the curve (AUC) values and 95% confidence intervals (CIs) were estimated by bootstrap resampling (1000 iterations). Differences between AUCs were evaluated using the DeLong test for correlated ROC curves. Correlation analyses between mtDNA-CN and telomere length were performed using Spearman’s rank correlation coefficient on log_10_-transformed values, both in the overall cohort and separately within HC and ET groups. Association analyses were conducted using linear regression models with log_10_-transformed TL as the dependent variable and log_10_-transformed mtDNA-CN as the main predictor. A crude model was first fitted, followed by an adjusted model including age, sex, and disease group as covariates. In addition, group-specific association analyses were performed separately in HC and ET subjects, adjusting for age and sex. Robust HC3 standard errors were used for regression models.

## Figures and Tables

**Table 1:** Demographic characteristics of the patient and control groups.

Characteristic	ET (*N* = 68)	HC (*N* = 62)	*p* Value
Age, median (Q1–Q3)	66 (56.8–73)	70 (65.2–75)	0.0086
Age at onset, median (Q1–Q3)	62 (56–71.25)	NA	NA
Sex, No. (%)			0.3332
Female	24 (35.29)	28 (45.16)	
Male	44 (64.71)	34 (54.84)	

Abbreviations: NA, not applicable; Q1, first quartile; Q3, third quartile.

**Table 2:** Association analyses between mtDNA-CN and TL. The adjusted model included age, sex, and disease group as covariates. β coefficients were estimated using linear regression models with log_10_-transformed telomere length as the dependent variable.

Model	Group	β Coefficient	Standard Error	95% CI	*p*-Value
Crude model	Overall	0.562	0.078	[0.410–0.714]	4.31 × 10^−13^
Adjusted model	Overall	0.510	0.101	[0.313–0.708]	3.93 × 10^−7^
	HC	0.529	0.112	[0.310–0.748]	2.21× 10^−6^
	ET	0.319	0.235	[−0.141–0.780]	0.174

## Data Availability

The data that support the findings of this study are available from the corresponding author (M.G.) upon reasonable request.

## Supplementary Materials

The following supporting information can be downloaded at: https://www.mdpi.com/article/10.3390/ijms27125275/s1. References [42,43] are cited in the supplementary materials.

## Abbreviations

The following abbreviations are used in this manuscript:

ET	Essential tremor
mtDNA-CN	Mitochondrial DNA copy number
TL	Telomere length
HC	Healthy controls
qPCR	Quantitative polymerase chain reaction
T/S	Telomere-to-single-copy
FDR	False discovery rate
*ACTB*	Actin beta
*SLC38A6*	Solute carrier family 38 member 6
GABA	γ-aminobutyric acid
*NOTCH2NLC*	Notch 2 N-terminal-like C
*HTRA2*	HtrA serine peptidase 2
MIQE	Minimum Information for Publication of Quantitative Real-Time PCR Experiments
CVs	Coefficients of variation
RQ	Relative quantification
HC3	Heteroskedasticity-consistent covariance matrix estimator type 3
ROC	Receiver operating characteristic
AUC	Area under curve
R^2^	Coefficient of determination
ΔΔCt	Delta–delta cycle threshold
β	Regression coefficient
CIs	Confidence intervals
*p*	*p* value
*ρ*	rho
Q1	First quartile
Q3	Third quartile
NA	Not applicable
mtDNA:nDNA	Mitochondrial DNA–nuclear DNA
IPMDS	International Parkinson and Movement Disorder Society
