# Supplementary material for: Reduced Mitochondrial DNA Copy Number and Telomere Length in Essential Tremor Patients: Evidence from an Age- and Sex-Adjusted Cross-Sectional Case–Control Study
Source: Int J Mol Sci. 2026 Jun 10;27(12):5275. doi: 10.3390/ijms27125275 (PMC13300436; doi:10.3390/ijms27125275)
Supplement: Supplementary file 1 [file ijms-27-05275-s001.zip › ijms-4331862-supplementary.pdf]

Supplementary File

Figure S1. *mtDNA-CN regression assumption assessment.* Diagnostic plots for mtDNA-CN regression model, including the Q-Q plot of residuals (left) and the residuals versus fitted values plot (right).

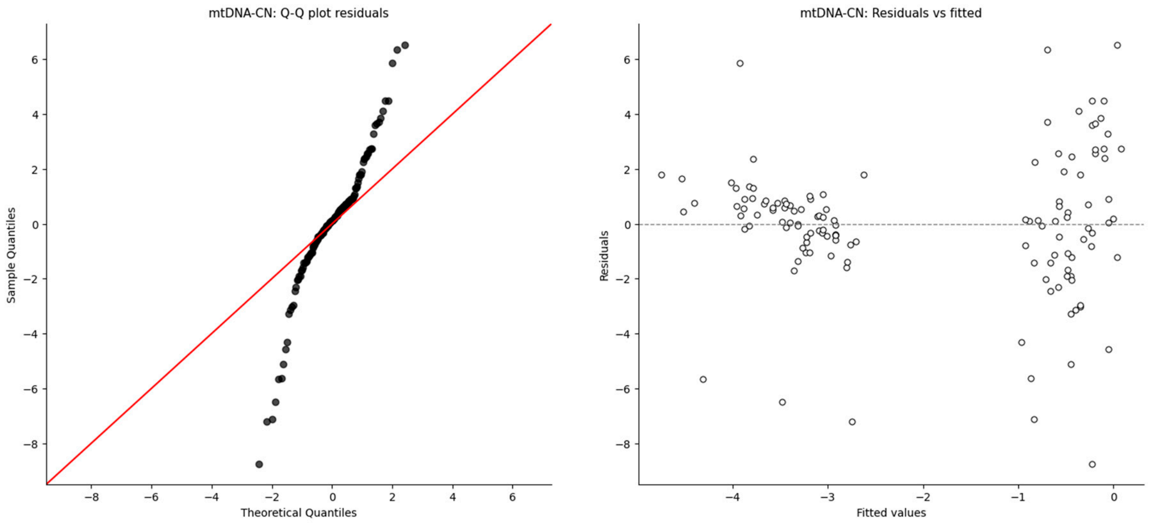

Figure S2. *Telomere length regression assumption assessment*. Diagnostic plots for telomere length regression model, including the Q-Q plot of residuals (left) and the residuals versus fitted values plot (right).

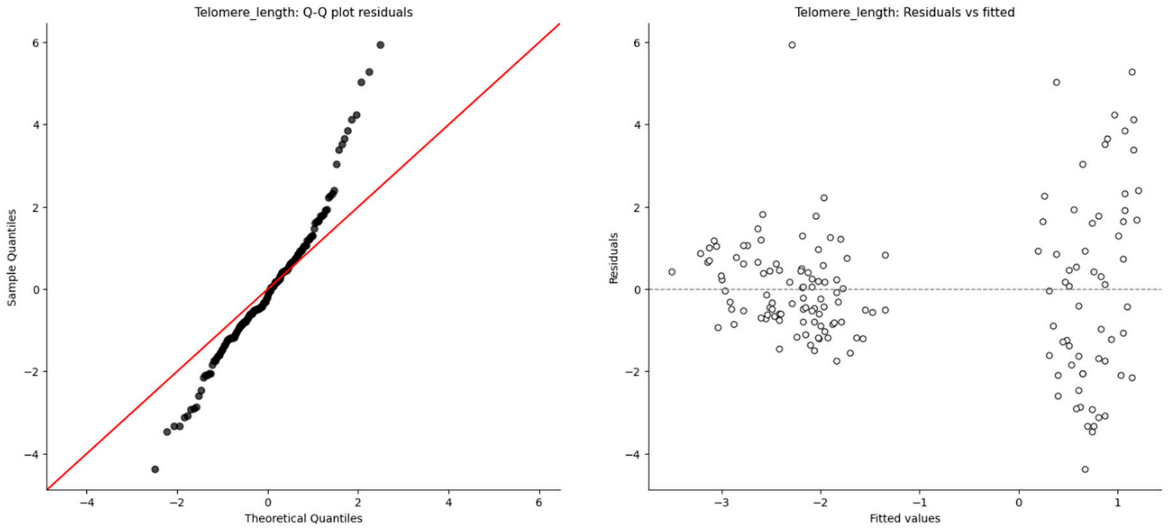

Table S1. *Primer sequences for mtDNA copy number and telomere length assays.* Primer sequences were adopted from previously published studies [1,2].

Primer	*Sequence (5′-3′)*	*Product (bp)*
MT-ND1_F	CACTCACATCACAGCGCTAA	
MT-ND1_R	GGATTATGGATGCGGTTGCT	
Telomere_F	CGGTTTGTTTGGGTTTGGGTTTGGGTTTGGGTTTGGGTT	
Telomere_R	GGCTTGCCTTACCCTTACCCTTACCCTTACCCTTACCCT	
b-actin_F	ATTGGCAATGAGCGGTTCCGC	543
b-actin_R	CTCCTGCTTGCTGATCCACATC	

Figure S3. *Standard curves for the β-actin, ND1 and telomere qPCR assays.* Five-point serial dilution curves used to assess primer performance and amplification efficiency for the quantitative PCR assays.

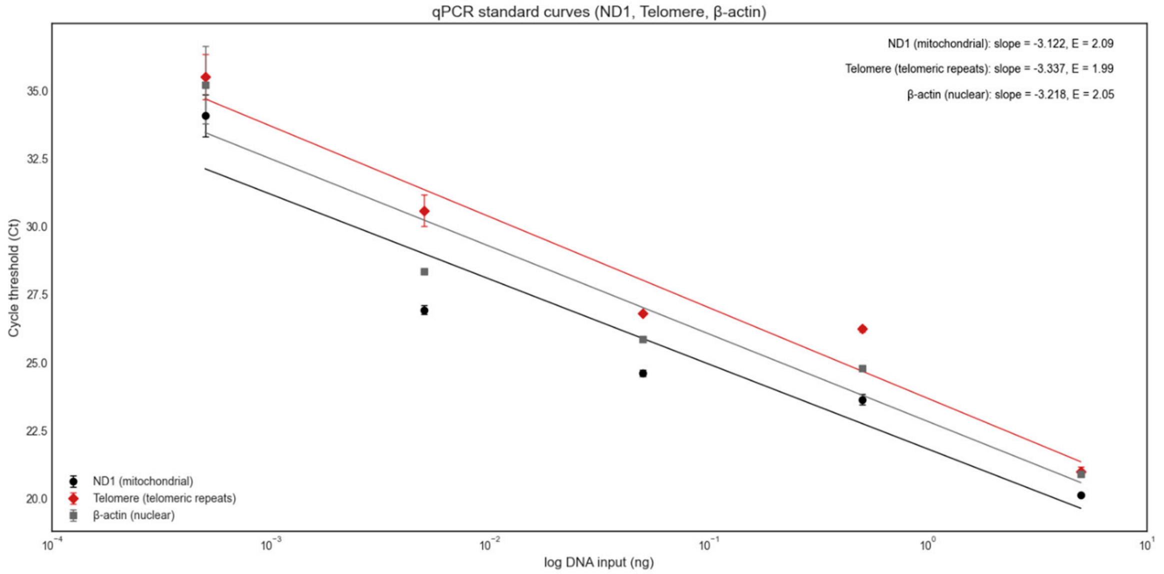

Table S2. STROBE Checklist for Cross-Sectional Studies.

Item	Section	STROBE recommendation	Location in manuscript / authors' response
**1a**	Title/Abstract	Indicate study design in title or abstract.	Reported in Title and Abstract.
**1b**	Title/Abstract	Provide balanced summary of what was done and found.	Reported in Abstract.
**2**	Introduction	Explain scientific background and rationale.	Reported in Introduction.
**3**	Introduction	State specific objectives and prespecified hypotheses.	Reported at end of Introduction.
**4**	Methods	Present key elements of study design early.	Reported as cross-sectional case-control study in section 4.1.
**5**	Methods	Describe setting, location, and relevant dates.	Reported in section 4.1 of Materials and Methods.
**6a**	Methods	Give eligibility criteria and selection methods.	Reported in section 4.1 of Materials and Methods.
**7**	Methods	Define outcomes, exposures, predictors, confounders/effect modifiers.	Primary outcomes were mtDNA-CN and telomere length; exposure was diagnostic group. Age and sex were included as covariates, and subgroup analyses were performed by age strata and sex.
**8**	Methods	Describe data sources and measurement methods; comparability between groups.	Mostly reported in Genetic analysis (4.2).
**9**	Methods	Describe efforts to address potential bias.	Sex matching, age/sex adjustment, standardized duplicate qPCR, QC CVs, robust SEs.
**10**	Methods	Explain how study size was arrived at.	Power/minimum detectable effect size.
**11**	Methods	Explain handling of quantitative variables and groupings.	Reported for $\log_2$ and $\log_{10}$ transformations.
**12a**	Methods	Describe statistical methods, including control for confounding.	Reported in section 4.3 of Materials and Methods.
**12b**	Methods	Describe subgroup/interactions methods.	Subgroup analyses were stratified by age group and sex.
**12c**	Methods	Explain how missing data were addressed.	No missing data, or exclusion/no imputation, according to the dataset.
**12d**	Methods	Describe sampling-strategy analytical methods, if applicable.	Not applicable.
**13a**	Results	Report numbers at each participant stage.	Partly reported: 68 ET and 62 HC.
**13b**	Results	Give reasons for non-participation.	Not applicable.
**13c**	Results	Consider a flow diagram.	Optional.
**14a**	Results	Give participant characteristics and confounders.	Reported in Table 1.
**14b**	Results	Indicate missing data for each variable.	No missing data for age and sex.
**15**	Results	Report outcome events or summary measures.	Reported for continuous biomarkers: estimates, CIs, RQ, AUCs, subgroup analyses.
**16a**	Results	Give unadjusted/adjusted estimates and precision.	Adjusted estimates/CIs reported. Adjusted and unadjusted estimates are reported for correlation analysis.
**16b**	Results	Report category boundaries.	Reported for age categories.
**16c**	Results	Translate relative risk to absolute risk if relevant.	Not applicable: continuous biomarkers, not incidence/risk analysis.
**17**	Results	Report other analyses.	Reported: age/sex strata, power, ROC.
**18**	Discussion	Summarize key results relative to objectives.	Reported in Results.
**19**	Discussion	Discuss limitations and potential bias/imprecision.	Reported extensively, including blood-cell composition and uncontrolled confounding.
**20**	Discussion	Give cautious interpretation.	Reported in Discussion.
**21**	Discussion	Discuss generalisability.	Discussed in section 3.3 of Discussion.
**22**	Other info	Give funding source and funder role.	Reported: no external funding.

References

1. Qin, L.; Huang, T.; Zhang, D.; Li, G.; Wei, L.; Liu, J. Elevated Mitochondrial DNA Copy Number in Euthyroid Individuals with Impaired Peripheral Sensitivity to Thyroid Hormones. *Front Endocrinol (Lausanne)* **2025**, *16*, 1635820, doi:10.3389/fendo.2025.1635820.
2. Cawthon, R.M. Telomere Measurement by Quantitative PCR. *Nucleic Acids Res* **2002**, *30*, e47, doi:10.1093/nar/30.10.e47.
